# Monodisperse measurement of the biotin-streptavidin interaction strength in a well-defined pulling geometry

**DOI:** 10.1371/journal.pone.0188722

**Published:** 2017-12-05

**Authors:** Steffen M. Sedlak, Magnus S. Bauer, Carleen Kluger, Leonard C. Schendel, Lukas F. Milles, Diana A. Pippig, Hermann E. Gaub

**Affiliations:** Lehrstuhl für Angewandte Physik and Center for NanoScience (CeNS), Ludwig-Maximilians-Universität München, Munich, Germany; National Institute for Medical Research, Medical Research Council, London, UNITED KINGDOM

## Abstract

The widely used interaction of the homotetramer streptavidin with the small molecule biotin has been intensively studied by force spectroscopy and has become a model system for receptor ligand interaction. However, streptavidin’s tetravalency results in diverse force propagation pathways through the different binding interfaces. This multiplicity gives rise to polydisperse force spectroscopy data. Here, we present an engineered monovalent streptavidin tetramer with a single cysteine in its functional subunit that allows for site-specific immobilization of the molecule, orthogonal to biotin binding. Functionality of streptavidin and its binding properties for biotin remain unaffected. We thus created a stable and reliable molecular anchor with a unique high-affinity binding site for biotinylated molecules or nanoparticles, which we expect to be useful for many single-molecule applications. To characterize the mechanical properties of the bond between biotin and our monovalent streptavidin, we performed force spectroscopy experiments using an atomic force microscope. We were able to conduct measurements at the single-molecule level with 1:1-stoichiometry and a well-defined geometry, in which force exclusively propagates through a single subunit of the streptavidin tetramer. For different force loading rates, we obtained narrow force distributions of the bond rupture forces ranging from 200 pN at 1,500 pN/s to 230 pN at 110,000 pN/s. The data are in very good agreement with the standard Bell-Evans model with a single potential barrier at *Δx*_*0*_ = 0.38 nm and a zero-force off-rate *k*_*off*,*0*_ in the 10^−6^ s^-1^ range.

## Introduction

**Fig 1:**
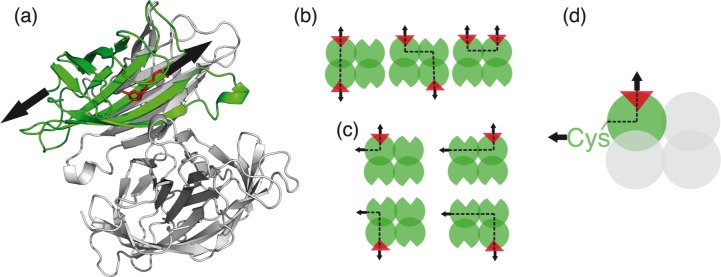
Possible pulling geometries for SA of different valencies. (a) Crystal structure of mSA (pdb identification code 5TO2 [15], overlaid with 1MK5 [16] to show the position of biotin). The functional subunit (green) with biotin (red) bound is stabilized by the three non-functional subunits (grey). Black arrows show the direction of the applied load for the AFM-based SMFS measurement. (b) Tetravalent SA consists of four functional subunits (green balls) each possessing a biotin (red triangles) binding site. In previous experiments, SA has been attached to a biotinylated surface resulting in a variety of possible pulling geometries: Across the strong interface, across the weak interface or diagonally across the tetramer. Having several functional binding pockets available, multiple binding to surface or cantilever can also occur. Black arrows indicate the pulling direction, black dotted lines possible ways force propagates through the molecule. (c) Attaching the tetravalent SA molecule covalently to the surface gives also rise to diverse pulling geometries. (d) In our experiments, we employ mSA consisting of one functional (green ball) and three non-functional subunits that are unable to bind biotin (grey balls). Having mSA tethered by a single N-terminal cysteine in the functional subunit, we pull biotin out of the binding pocket. The force only propagates through a single subunit.

With its low dissociation constant in the femtomolar range [1], its specificity, and its high stability under harsh conditions [2], the binding of the small molecule biotin to the homotetramer streptavidin (SA) is a popular and widely used tool in nanotechnology, biotechnology, and medicine. Especially after biotinylation became available [3], this receptor-ligand system found versatile applications, e.g. detection [4, 5] or capturing of biomolecules [6–9], and diverse other *in vivo* and *in vitro* methods. For single-molecule techniques, the tetravalency of SA can however be disadvantageous, as it promotes clustering of biotinylated molecules. Single-molecule force spectroscopy (SMFS) [10], super-resolution imaging techniques, and analytical applications like surface plasmon resonance or switch sense technology [11] often require a 1:1 stoichiometry. Efforts have been directed at the development of monomeric versions of SA [12]. However, since the interplay between different subunits is important for the tight binding of biotin [13], monomeric SAs lack the outstanding affinity of wildtype SA [12]. In 2006, Howarth et al. [14] developed a tetrameric but monovalent streptavidin (mSA), by reconstituting one functional with three non-functional subunits (Fig 1A). mSA preserves femtomolar affinity towards biotin. Here, we present the implementation of mSA as a molecular anchor for atomic force microscopy (AFM)-based SMFS, which enables us to revisit the biotin:SA interaction in a very specific and monodisperse manner.

The interaction between biotin and tetravalent SA/avidin was the first receptor-ligand interactions probed by AFM-based SMFS [17–19]. It has become a model system for non-covalent receptor-ligand complexes and to study biorecognition processes [20]. In an AFM-based SMFS measurement, a functionalized AFM-cantilever decorated with ligand molecules is approached to a functionalized surface decorated with receptor molecules. A receptor-ligand complex is formed and when retracting the cantilever from the surface, the bending of the cantilever is recorded providing a measure for the force that the receptor-ligand complex can withstand, i.e. for its mechanical strength under load.

In 1994, Moy et al. [19] reported integer multiples of biotin:SA unbinding events and analyzed the relation between binding energies and unbinding forces. Biotinylated bovine serum albumin (BSA) was unspecifically adsorbed to both cantilever and sample surface. Bringing cantilever and surface in contact, SA that had been added to the solution could bind to a biotin on the cantilever and to one on the surface at the same time. Retracting the cantilever from the surface, the force needed to pull biotin and SA apart was recorded. The way load was applied to tetravalent SA in this experiment is schematically described in Fig 1B. Combinations of the geometries shown in this figure are also likely to occur. To obtain data at the single-molecule level, either the concentration of SA molecules was adjusted or free biotin was added to the solution.

Several groups independently repeated the experiment [18, 21]. Allen et al. slightly modified the setup by direct, yet unspecific, immobilization of SA to the sample surface [22]. In the following years, the biotin:SA interaction was modeled by MD simulations [23, 24] and theoretical descriptions for the process of unbinding were put forward [25–27]. In 1999, Merkel et al. [28] measured the biotin:SA interaction with a biomembrane force probe instrument. For the first time, measurements using different force loading rates were performed. On top of that, they introduced covalent attachment of biotin through polyethylene glycol (PEG) linkers. With a covalent immobilization strategy, detachment of biotin from the sample surfaces became unlikely, resulting in higher purity of the recorded data. The variety of possible pulling geometries, as depicted in Fig 1B, remained. Using the loading-rate dependence of rupture forces, the energy landscape of the biotin:SA binding was investigated. Dynamic force spectra of the receptor-ligand system were also recorded with the AFM using diverse attachment strategies, such as immobilization in a phospholipid bilayer [29] or a dextran-coated surface [30], by biotinylated BSA [31–33] or by cross-linking with glutaraldehyde [34]. In 2010, Taninaka et al. further improved the measurement procedure by binding both biotin and SA covalently with PEG spacers to sample and cantilever surface, respectively [35]. The way load is applied to the SA tetramer in this case is shown in Fig 1C.

Due to different ways the ligand binds to the receptor, AFM-based SMFS data can be dispersed when performing experiments using multivalent receptor molecules, such as SA, even if actual single-molecule interactions are probed. Pulling on the ligand, the force can propagate through the receptor molecule in different ways (Fig 1B and 1C). This results in a broad distribution of rupture forces. Furthermore, when the receptor molecule is composed of several non-covalently bound subunits, the data are distorted if the subunits of the receptor molecule get torn apart. In a SMFS experiment, a rupture of the receptor molecule itself cannot be distinguished from the unbinding of the ligand from the receptor. Beyond that, disrupted receptor tetramers may clog the cantilever thus preventing specific interaction resulting in low data yield.

From the crystal structure of wild-type SA, it can be reasoned that the SA monomers assemble into strongly associated dimers that form less stable tetramers [36]. Therefore, the different interfaces between the four subunits of a SA tetramer might be of different mechanical stability. Kim et al. [37] proved that the mechanical strength of the SA tetramer itself is highly dependent on the pulling geometry, i.e. on the way force is applied to the tetramer. Pulling on various control domains that were genetically fused to the N-termini of the SA monomers, they observed two distinct peaks in the distribution of rupture forces of the tetramer [37]. The two peaks can be assigned to a rupture across the strong interface between two subunits forming a dimer and to the rupture across the weak interface between the two dimers forming the tetramer. Interestingly, the force peaks of around 100 pN and 400-500 pN overlap with the range of unbinding forces reported for the biotin:SA interaction [18, 19, 21, 22, 28–32, 35, 38–40].

Non-equilibrium unbinding forces are loading rate dependent [41]. Any comparison of unbinding forces on an absolute scale, especially when measured with different setups under different conditions, is to be treated with caution. Nevertheless, it is conceivable that SMFS experiments with biotin and tetravalent SA are to some extend distorted by the potential rupture of the tetramer before unbinding biotin from SA. To examine the behavior of the biotin:SA interaction under load, it is therefore important to overcome the problem of SA’s tetravalency.

We therefore implement mSA to perform high-throughput AFM-based SMFS experiments for probing the mechanical stability of the biotin:SA system in a well-defined pulling geometry, no longer distorted by the receptor’s multivalency. The quality of the data is further improved by the use of protein calibration domains for identification of single interactions. The unfolding patterns of the calibration domains that are enzymatically fused to ligand or receptor molecule verify single rupture events. When unfolding under the applied load before the receptor-ligand complex ruptures, they yield a specific unfolding force, which serves as internal reference for force calibration, and a defined length increment that is taken as an indicator for single receptor-ligand unbinding.

For site-selective immobilization of SA, we genetically modified the functional subunit of mSA. Although wildtype SA does not contain any cysteine residues, the SA tetramer was found to be of high stability under conditions, which are usually denaturing [42]. In contrast to many other proteins, the interaction between the subunits is not mediated by disulfide bridges but originates from a network of hydrogen bonds and hydrophobic interactions. We thus introduced a single cysteine at the N-terminus of the functional subunit of mSA for site-selective immobilization by conventional thiol-maleimide coupling [43]. We thereby created a stable molecular anchor for biotinylated (bio-)molecules with femtomolar affinity and well-defined stoichiometry. This well-defined single anchor point together with the monovalency of the biotin mSA interaction defines an unambiguous force propagation path. It enables us to perform AFM-based SMFS experiments in which the force only propagates through a single subunit of SA (Fig 1D).

## Materials and methods

### Gene construction, protein expression and purification

A detailed description of expression and purification is provided in the supplement (S1 Appendix). SA and mutant SA (deficient in biotin binding) constructs containing an N-terminal polyhistidine-tag (His-tag) for purification were cloned into pET vectors (Novagen, EMD Millipore, Billerica, USA). Constructs contained an N-terminal cysteine for site-specific immobilization, except for the subunits that were not meant to attach to AFM-cantilever surface or the glass coverslip. SA subunits with and without cysteine and His-tag and mutant SA subunits were expressed separately in *E*. *coli* BL21(DE3)-CodonPlus (Agilent Technologies, Santa Clara, USA). The constructs formed inclusion bodies that were isolated as described previously [44]. To reconstitute mSA and to guarantee a 1:3 ratio of functional to non-functional SA subunits in the final tetramer, inclusion bodies were solubilized in 6 M guanidine hydrochloride and then mixed at a 1:10 ratio prior to refolding and purification via the His-tag. To obtain tetravalent SA with a unique cysteine coupling site, the construct containing the cysteine residue as well as a His-tag was mixed with functional SA devoid of either.

The *Dictyostelium discoideum* fourth filamin domain (ddFLN4) construct with an N-terminal ybbR-tag [45] and a C-terminal cysteine (the internal cysteine 18 was mutated to serine) was cloned into pET vectors (Novagen, EMD Millipore, Billerica, USA). After expression in *E*. *coli* BL21(DE3)-CodonPlus (Agilent Technologies Santa Clara, USA) and lysis, purification was achieved by immobilized metal ion affinity chromatography (Ni-IMAC).

The superfolder green fluorescent protein (GFP) construct with an N-terminal cysteine and a C-terminal ybbR-tag was cloned into pET vectors (Novagen, EMD Millipore, Billerica, USA) and expressed in *E*. *coli* BL21(DE3)-CodonPlus (Agilent Technologies Santa Clara, USA). Purification was performed by Ni-IMAC.

### Biotinylation of protein constructs

GFP and ddFLN4 constructs were biotinylated using the ybbR-tag/Sfp-Synthase system [45]. For the GFP construct, 18 µM GFP-ybbR were incubated with 60 µM CoA-Biotin (New England BioLabs) and 9 µM Sfp Synthase in a solution of 10 mM MgCl_2_ and 50 mM HEPES at pH 7.5 for 1 h at 37°C. To clean the solution from remaining CoA-Biotin, a buffer exchange to phosphate buffered saline (PBS; Sigma-Aldrich, Saint Louis, USA) was performed with Zeba Spin Desalting Columns (Thermo Scientific, Rockford, USA) with 7K MWCO according to the manufacturer’s instructions. For the ddFLN4 construct, the incubation was performed at room temperature. All other steps were done in the same way as for GFP.

### SDS-PAGE

Gel electrophoresis was performed using Any kD Mini-PROTEAN TGX Precast Protein Gels (Bio-Rad, Hercules, USA) in TRIS-based running buffer (2.5 mM TRIS, 200 mM glycerol, 3.5 mM SDS). For lanes 2–4, we heated 0.6 µM SA dissolved in loading buffer (50 mM TRIS, pH 8.0, 2.5% SDS, 5% glycerol, 0.005% bromophenol blue, 2.5% β-mercaptoethanol) for 5 minutes to 95°C. For the other SA containing lanes, we used about 1.5 µM. For lanes 10–13, we added 1 µl of the purified Sfp reaction mixture containing both biotinylated and un-biotinylated GFP. We employed Precision Plus Unstained Protein Standards (Bio-Rad Laboratories, Hercules, USA) as molecular weight standards. The gel was run at room temperature with a constant current of 25 mA. The gel was analyzed with a ChemiDoc MP Imaging System (Bio-Rad Laboratories, Hercules, USA).

### Isothermal titration calorimetry

The calorimetric experiments were carried out with a Malvern MicroCal ITC200 (Malvern, UK). SA samples were equilibrated with PBS using Zeba Spin Desalting Columns (Thermo Scientific, Rockford, USA) with 40K MWCO following the manufacturer’s instructions. The concentration was determined by spectrophotometry with a NanoDrop 1000 (Thermo Scientific, Rockford, USA) using an extinction coefficient of ε_280_ = 167,760 M^-1^cm^-1^ calculated from the protein sequence using the SIB bioinformatics resource portal [46]. Biotin (Sigma-Aldrich, St. Louis, USA) was dissolved in PBS. For all measurement, the same stock solution of biotin was used. For mSA, a tenfold excess of biotin was titrated into the sample cell. For tetravalent SA, we used a ratio of 1:40, resulting in a final molar ratio of 1:8. All experiments were performed at 25°C.

### Functionalization of cantilevers and coverslips

AFM cantilevers (Biolever Mini, Olympus, Tokyo, Japan) and glass coverslips were silanized as described by Zimmermann et al. [43]. They were incubated with 25 mM heterobifunctional PEG (Rapp Polymere, Tübingen, Germany) with a molecular weight of 5 kDA equipped with an N-Hydroxysuccinimide (NHS) group and a maleimide group dissolved in a 50 mM HEPES solution at pH 7.5 for 45 minutes. The PEG spacers ensure passivation of glass cover slip and AFM-cantilevers and allow for specific sample immobilization. The coverslips were washed in ultrapure water and mounted into AFM sample holder. A 3.5 µl droplet of monovalent or tetravalent SA was deposited on the surface. The cantilevers were washed in ultrapure water and then placed in a 15 µl drop of the purified biotinylated ddFLN4 construct. For an efficient reaction of thiol with maleimide groups which forms stable thioester bonds, we reduced the thiol groups of SA and ddFLN4 construct by adding Immobilized TCEP Disulfide Reducing Gel (Thermo Scientific, Rockford, USA) in a v/v ratio of 1:6 and incubated for 1 h. The gel was removed with the help of an Ultrafree-MC, HV 0.45 µm centrifugal filter (Merck Millipore, Darmstadt, Germany) directly before adding the proteins to the coverslips or cantilevers. During the formation of the thioester bonds, the samples were kept in a humidity chamber to prevent evaporation. After 1.5 h, the cantilevers were washed twice in PBS and the surfaces were rinsed with 50 ml PBS to flush out unbound protein.

### AFM-based single-molecule force spectroscopy experiments

The experiments were performed with a custom-built AFM as described by Gumpp et al. [47]. The cantilevers were approached to the surface and after short contact, retracted at constant velocities of 200 nm/s, 800 nm/s, 2,000 nm/s, 5,000 nm/s, and 10,000 nm/s. To always probe a different spot on the surface, it was horizontally moved by 100 nm after each approach. For calibration of the cantilevers, we employed the equipartition theorem [48]. Baumann et al. [44] and Milles et al. [49] provide detailed descriptions of experimental SMFS procedures and SMFS data analysis.

## Results and discussion

### Size and functionality of mSA constructs with terminal cysteine is maintained

**Fig 2:**
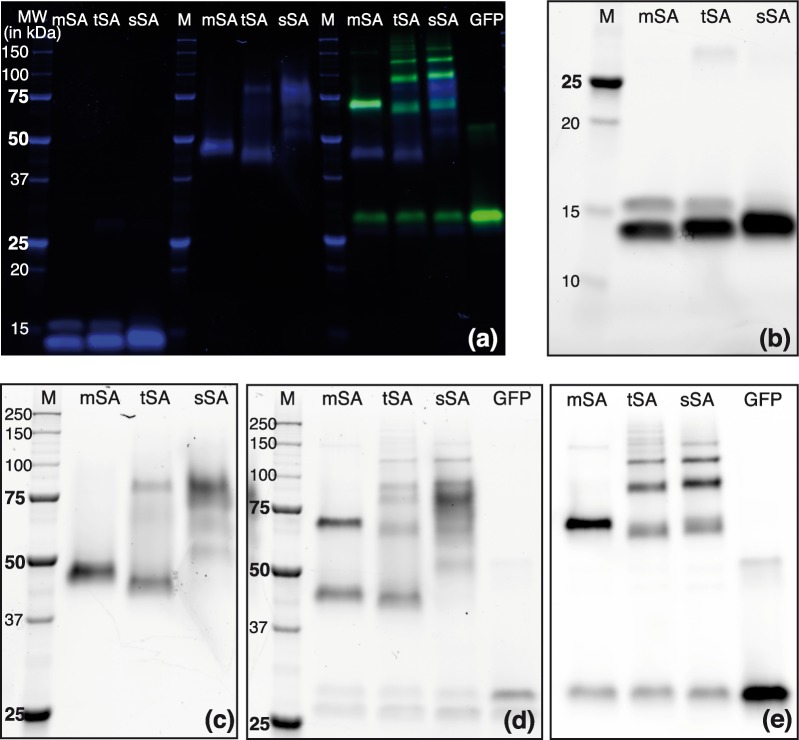
SDS-PAGE of mSA, tSA and commercial SA from *streptomyces avidinii* (sSA). (a) Overview of differently treated SAs with and without addition of biotinylated GFP on a stain-free polyacrylamide gel. Overlay of images taken with UV light excitation (blue) and illumination with a blue LED source (green). Parts of this image are inverted and shown in detail (b-d UV-excitation; e: GFP-channel): (b) Denatured SA samples (5 min at 95°C). Decomposition into monomers (14 kDa) is visible. His-tagged subunits appear larger. sSA subunits are smeared out. (c) Untreated SA samples which maintain tertiary structure. (d,e) Addition of biotinylated GFP to untreated SA samples. Valencies of SAs are visible as different numbers of GFPs are bound. The lowest band in (d) corresponds to Sfp Synthase (26 kDa).

After expression and purification, we checked size and quality of the SAs with SDS polyacrylamide gel electrophoresis (Fig 2). Heating mSA and tetravalent SA (tSA) for 5 min to 95°C, the tetramers fall apart into monomers of approximately 14 kDa (Fig 2B). The higher band can be assigned to the monomer with the additional His-tag and we confirmed the expected ratio between the monomers to be 1:3. Commercially available SA from *Streptomyces avidinii* (sSA) shows only one slightly larger and broader band. In contrast to the recombinantly expressed core SA monomer that consist of 123 residues, the SA monomer from *Streptomyces avidinii* contains 183 amino acids. In a posttranslational digest process, it is cut down to core SA.

The size of the tetramers can be estimated from unheated samples (Fig 2C). For mSA and tSA band size is slightly below the expected 54 kDa. Bands at double size are attributed to two tetramers connected via disulfide bridges between their cysteine residues. sSA shows several smeared out bands of larger size, caused by an incomplete posttranslational digest. The lowest one corresponds to core SA (54 kDa).

To illustrate the binding stoichiometry of the SAs to biotin, we added biotinylated GFP to mSA, tSA, and sSA (Fig 2D and 2E). Since the biotinylation of GFP has been incomplete, bands of unbound SA and bands of GFP without biotin are still visible. All SAs having a single GFP bound appear at the same size of about 70 kDa. Valencies of the different SA can be determined from the number of bands. For mSA, only one band with a single biotinylated GFP bound is seen. For sSA, four bands are clearly visible. Because of dimerized tetramers binding one or several biotinylated GFPs, additional bands appear for tSA.

### Modifications of mSA do not change biotin binding properties

**Fig 3:**
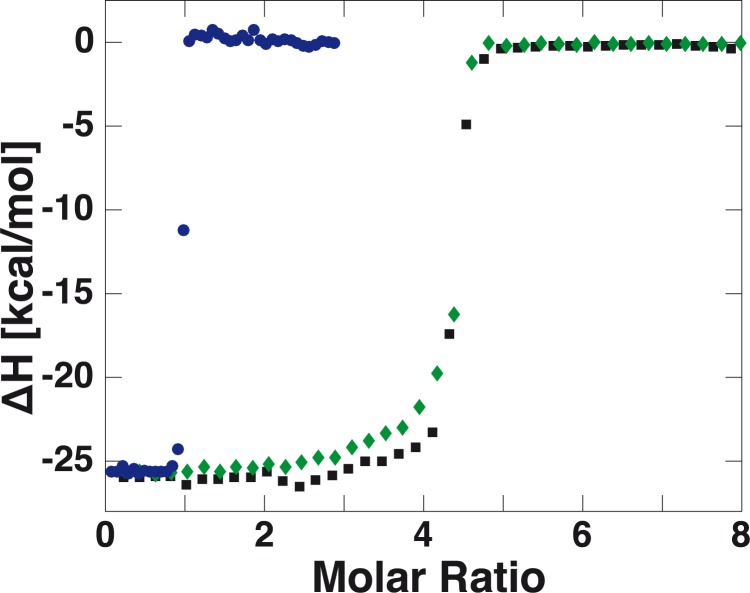
Isothermal titration calorimetry of biotin and SAs of different valency. The binding of biotin to different SAs was measured with isothermal titration calorimetry. The binding stoichiometry of mSA and biotin was determined as *N* = 0.95 (blue circles). The measured binding stoichiometry of the engineered tetravalent version (green diamonds) *N* = 4.31 is in good agreement with the value of commercial SA isolated from *Streptomyces avidinii* (black squares) *N* = 4.29. Within the limits of the measurement’s accuracy, the binding enthalpies of the different SAs are the same (*ΔH* = -26 kcal/mol for monovalent, *ΔH* = -25 kcal/mol for tetravalent and *ΔH* = -26 kcal/mol for commercial SA), confirming that the N-terminal modifications do not interfere with the binding of biotin.

We compared the binding properties of our modified mSA with tSA and sSA by isothermal titration calorimetry (Fig 3). Because of the high affinity of biotin to SA, we could only conclude that the dissociation constant *K*_*D*_ is lower than 1 nM. The binding enthalpy per mole of added biotin (*ΔH*_*mSA*_ = -26 kcal/mol, *ΔH*_*tSA*_ = -25 kcal/mol, *ΔH*_*sSA*_ = -26 kcal/mol) and the binding stoichiometry (*N*_*mSA*_ = 0.95, *N*_*tSA*_ = 4.31, *N*_*sSA*_ = 4.31) confirmed that the functional subunit of our modified mSA is capable of binding biotin in the same manner as the subunits of sSA, while the binding of biotin to the mutated non-functional subunits is negligible. The measured enthalpies are also in line with previously reported values [50]. This implies that the modifications at the N-terminus of the functional subunit do not impede the binding of biotin. We therefore argue that structure and function of the sSA are preserved for our monovalent and tetravalent versions with N-terminal modifications.

### AFM-based SMFS using mSA as a handle

**Fig 4:**
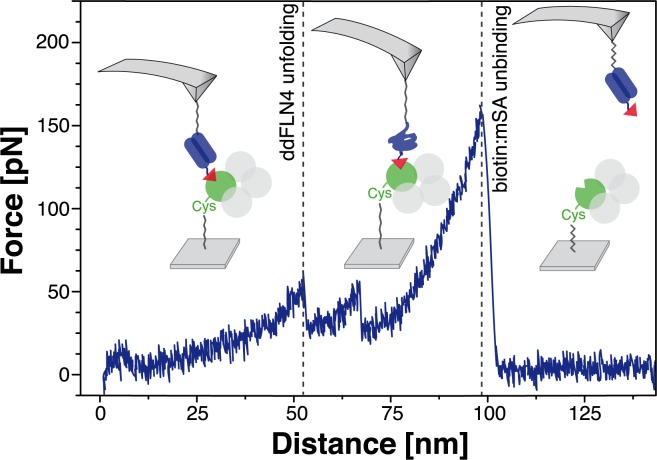
Investigation of the mechanical stability of the biotin:mSA binding with a well-defined pulling geometry. The functionalized cantilever tip is approached to the surface and a bond between biotin (red triangle) and mSA (green and gray balls) is formed. First, only the PEG (grey lines) spacers are stretched, when retracting the cantilever with constant speed from the surface. At forces of about 60 pN, the ddFLN4 (blue) unfolds in a characteristic two-step process that is used to identify single-molecule interactions. PEG spacers and the polypeptide chain are then further stretched until biotin unbinds from mSA under the applied load. The force drops and ddFLN4 folds back into its native state. As an example, one of the recorded force-distance curves (pulled at 800 nm/s) is shown in blue. More force-distance curves are shown in the supplement (S2 Appendix).

Using reconstituted mSA in combination with a calibration domain, we were able to perform SMFS with a well-defined pulling geometry that are not distorted by SA’s multivalency. In our experiments, force propagates only through a single subunit of the SA tetramer (Fig 1D). Therefore, no tension across any interface within the tetramer, which could cause dissociation of the tetramer into its subunits, is applied. The measurement process is illustrated in Fig 4. To ensure the specificity of the probed interaction, we used the unfolding pattern of biotinylated ddFLN4 [51] to identify single molecule rupture events. Because ddFLN4 folds back into its native state when the force drops after unbinding of biotin from mSA, it was used as a calibration domain on the cantilever, while mSA was immobilized on the surface. We use this attachment strategy for probing the biotin:mSA interaction, because we can probe a new mSA molecule, which has not yet been exposed to pulling forces, for every force-distance curve. Only those force curves that showed the specific unfolding pattern of the calibration domain were considered in subsequent data analysis procedures.

### Analysis of AFM-based SMFS data

**Fig 5:**
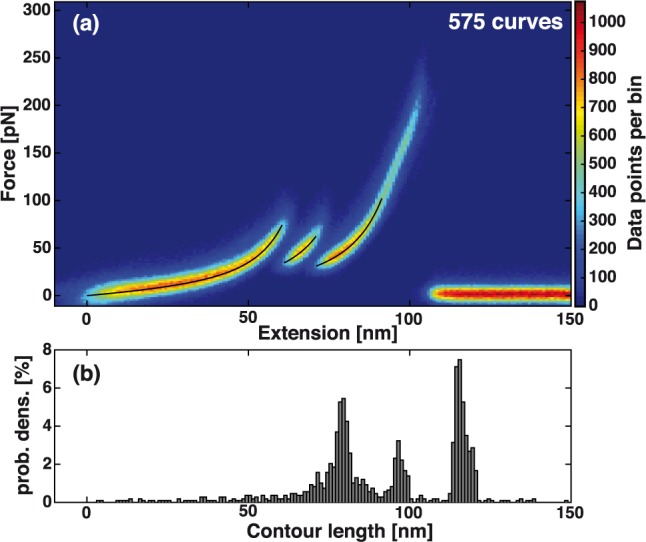
Overlay of force-extension curves and transformation into contour length space. (a) The 575 force-extension curves for which the characteristic unfolding pattern of ddFLN4 was visible are overlaid. We fit the three parts of the curve independently with the worm-like chain polymer model (black lines). (b) Using the mean persistence length of the worm-like chain fits, each point of the force extension curve is translated into contour length space. From the histogram, the contour lengths of the stretched constructs corresponding to the three parts of the force curve are determined.

In an AFM experiment, about 5,000 force extension traces were recorded of which about 1,100 showed interaction. A larger data set of over 50,000 traces obtained in a 15 h measurement is shown in the supplement (S3 Appendix). To prove reliability and reproducibility of the control domain’s unfolding pattern, an overlay of all 575 force-distance curves that feature the distinct unfolding pattern of ddFLN4 before biotin unbinds from mSA is shown in Fig 5A.

For every data bin along the extension axis, we selected the force bin with the highest value to obtain a characteristic force-extension curve. The curve consists of three parts: First, only the PEG-spacers on the cantilever and the surface are stretched (Fig 4). Then ddFLN4 unfolds in two distinct steps. Using the worm-like chain model for semi-flexible polymers [52] to fit this characteristic curve (black lines in Fig 5A), we deduced persistence lengths and contour lengths of the stretched construct for the different unfolding steps of the calibration domain. As the PEG-spacers undergo a conformational transition from cis to trans above forces of about 100 pN [53, 54] resulting in a linear force extension relation, we restricted the WLC fit to the part of the curve with forces lower than 100 pN. We find persistence lengths of 0.240 nm for the PEG-stretch, 0.265 nm and 0.282 nm for the subsequent parts. The fitted contour lengths of 80.7 nm, 96.4 nm, and 113.5 nm are in good agreement with theoretical estimations. From the molecular weights, we estimated the lengths of the two PEG-spacers to be about 31 nm to 40 nm each and the total contour length increment resulting from ddFLN4 unfolding to be 36 nm (S4 Appendix).

From the worm-like chain model, an expression for the contour length as a function of persistence length, force and extension can be derived [55]. Assuming a constant persistence length of 0.26 nm, we translated every data point of the characteristic curve (Fig 5A) into contour length space (S5 Appendix). In Fig 5B, the corresponding histogram of contour lengths is shown. Three pronounced peaks with maxima at 79.5 nm, 96.5 nm and 113.5 nm are visible, confirming the correct assignment of the different parts of the force-extension curve to different parts of our molecular construct.

**Fig 6:**
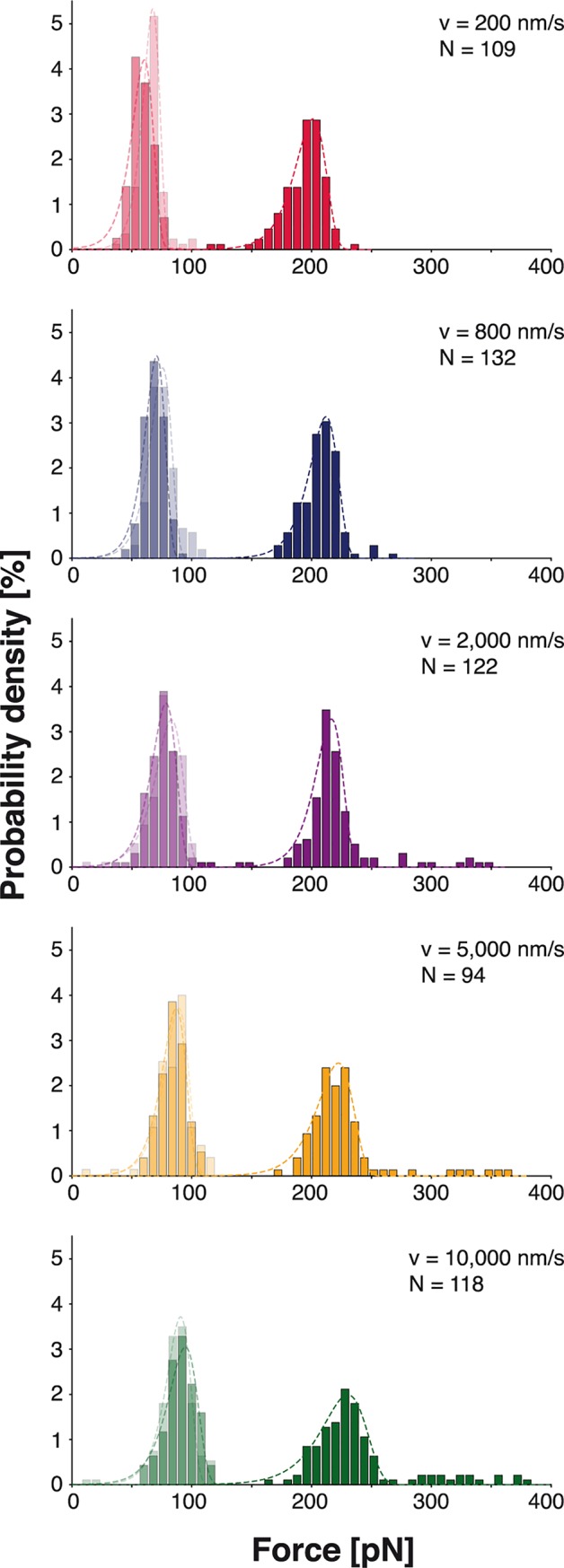
Unfolding forces of ddFLN4 and unbinding forces of biotin and mSA for different pulling velocities. The distribution of the forces of the first (transparent bars in the background) and second (semi-transparent bars) step of the ddFLN4 unfolding gives rise to two distinct peaks at approximately 85 pN and 75 pN. The biotin:mSA unbinding forces (opaque bars) are distributed more broadly but exhibit a clear maximum at about 200 pN depending on the applied force loading rate. The experiment was carried out with a cantilever with a spring constant of 73.9 pN/nm. The dashed lines show independent fits of Bell-Evans distributions to the force histograms.

We probed the biotin:mSA complex with five different retraction velocities (200 nm/s, 800 nm/s, 2,000 nm/s, 5,000 nm/s and 10,000 nm/s). The distributions of the resulting forces of the biotin:mSA unbinding and the ddFLN4 unfolding are depicted in Fig 6. The histograms of the forces corresponding to the two subsequent ddFLN4 unfolding steps exhibit defined peaks at 60-80 pN. For biotin:mSA unbinding force histograms, a sharp peak at about 200 pN is found. Its exact position depends on the applied loading rate. To obtain exact values, all force histograms were fitted with Bell-Evans models [25, 41] yielding the most probable rupture force, off-rates and distance to the transition state (S6 Appendix).

**Fig 7:**
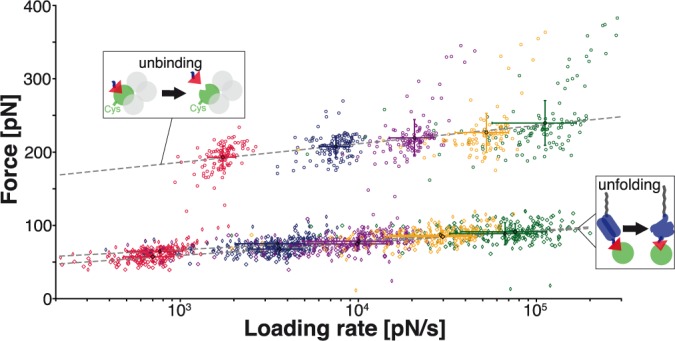
Bell-Evans plot of unfolding and unbinding forces. For all specific single-molecule interactions, the unbindig forces of biotin:mSA (circles) and the forces of the first (diamonds) and second (squares) step of the ddFLN4 unfolding are plotted against the loading rates at the corresponding force peak. The data are equal to the one shown in Fig 6 and the same color code is used. The dashed lines are linear fits to the centers of gravity (shown as filled circles, diamonds and squares) of the distributions of forces and loading rates, respectively. The colored crosses indicate the corresponding standard deviations.

The dynamic force spectrum is shown in Fig 7. Force loading rates were determined by fitting a linear slope over the last 3 nm before unfolding and unbinding force peaks in the force-extension curves. In the semi-logarithmic plot, the centers of gravity of force and loading rate distributions for the ddFLN4 unfolding and the biotin:mSA unbinding are fitted by a straight line. This linear dependence of unfolding or rupture forces on the loading rate is given by Bell-Evans theory (S5 Appendix). From slope and y-intercept, the distance to transition state *Δx*_*0*_ and the zero-force off-rate *k*_*off*,*0*_ can be determined. For the ddFLN4-unfolding, we find *Δx*_*0*_ = (0.76 ± 0.05) nm and *k*_*off*,*0*_ = 8 × 10^-4^ s^-1^ for the first unfolding peak and *Δx*_*0*_ = (0.56 ± 0.02) nm and *k*_*off*,*0*_ = 5 × 10^-2^ s^-1^ for the subsequent peak. The distance to the transition state of the biotin:mSA unbinding reads *Δx*_*0*_ = (0.38 ± 0.02) nm and the zero-force off-rate is determined as *k*_*off*,*0*_ = 3 × 10^-6^ s^-1^. The off-rate is in good agreement with the value obtained in an off-rate assay (*k*_*off*,*exp*_ = 6.1 × 10^−5^ s^-1^) [14]. Previous studies reported a kink in the force-loading rate dependence that was attributed to two potential barriers in the binding potential [28]. For the range of loading rates we applied and for the specific geometry that we used to load the complex, we could not observe this feature.

## Conclusion

Even though binding of biotin to SA is widely used as a tool and has been extensively studied previously, the unbinding forces reported in the literature scatter substantially. With the development of mSA and progress in AFM-based SMFS it became possible to study the mechanical stability of the biotin:SA complex in a better defined way. Relating to previous measurements of the unbinding of biotin from tetravalent SA, we illustrated how multivalency of receptor molecules can distort SMFS data of receptor-ligand unbinding. We presented AFM-based SMFS data of the unbinding of biotin from monovalent SA with a 1:1-stoichiometry in a distinct pulling geometry, in which the force only propagated through a single subunit of the SA tetramer. The main improvements of our measurements contributing to the high quality of our data are covalent immobilization of both receptor and ligand molecules, the use of a calibration domain to verify single-molecule interaction events, and exact control over the attachment geometry by a single distinct anchoring site and monovalent receptor molecules.

Beyond that, we introduced a new tethering strategy for the use of mSA not only in force spectroscopy but also in many other single-molecule applications. The immobilization of mSA by implementing a single cysteine at the terminus of the functional subunit provides an anchoring site for sulfhydryl-reactive chemical groups, i.e. an anchoring site that is orthogonal to the interaction with biotin. In contrast to defined divalent SA [56] that can serve as a molecular hub for biotinylated molecules, mSA engineered with a single terminal cysteine on the functional subunit allows for controlled immobilization of biotinylated biomolecules or nanoparticles providing a 1:1-binding site.

## Supporting information

S1 Appendix Streptavidin preparation. (PDF)

S2 Appendix Exemplary force-distance curves. (PDF)

S3 Appendix Long-term SMFS measurement. (PDF)

S4 Appendix Estimating the contour lengths of PEG and ddFLN4. (PDF)

S5 Appendix Formulas. (PDF)

S6 Appendix Fitted Bell-Evans distributions shown in Fig 6. (PDF)

S7 Appendix Sequences of protein constructs. (PDF)

S8 Appendix Measuring with mSA immobilized on the cantilever. (PDF)
