# Supplementary material for: Monodisperse measurement of the biotin-streptavidin interaction strength in a well-defined pulling geometry
Source: PLoS One. 2017 Dec 5;12(12):e0188722. doi: 10.1371/journal.pone.0188722 (PMC5716544; doi:10.1371/journal.pone.0188722)
Supplement: S1 Appendix — (PDF) [file pone.0188722.s001.pdf]

S1 Appendix.

Streptavidin preparation

Streptavidin Cloning

SA variants were obtained by site-directed mutagenesis of plasmids encoding Strep-Tactin constructs, whose sequence is similar to streptavidin [1], using a polymerase chain reaction and subsequent blunt-end ligation. By DNA sequencing (Eurofins Genomics, Ebersberg, Germany), we checked all final open reading frames.

Streptavidin Expression

The different SA subunits were expressed separately in *E.coli* BL21(DE3)-CodonPlus cells (Agilent Technologies, Santa Clara, USA). Plasmids encoding for different SA constructs, were transferred into *E.coli* BL21(DE3)-CodonPlus cells. Cells were grown at 37°C in pure LB Medium to build up antibiotic resistance, spread on an agar plate containing the appropriate antibiotic, and grown for 18 h at 37°C. We inoculated a preculture (8 ml LB medium, 1:1000 antibiotic) and grew the cells for 15 h at 37°C. We added preculture to the expression medium (500 ml SB medium with 20 mM KH_2PO_4 and 1:1000 antibiotic) until an optical density (absorbance at 600 nm) $OD_{600} = 0.1$ was reached. The expression culture was grown at 37°C until the optical density read $OD_{600} = 0.8$. After adding 1:5000 IPTG, the culture was grown for 15 h at 18°C. Then, it was centrifuged at 24,000 × g for 15 min. A bacterial pellet formed and was stored at -80 °C.

Streptavidin Purification

During all steps, samples were kept at 4 °C or on ice, respectively. Bacterial pellets for functional and non-functional subunits were weighed and then lysed separately in 5 ml Bacterial Protein Extraction Reagent (B-PER; Thermo Scientific, Rockford, USA) per gram bacterial pellet. We added 1 mg Lysozyme (Carl Roth GmbH & Co. KG, Karlsruhe, Germany) and 50 μg DNase I (Roche Diagnostics GmbH, Mannheim, Germany) per gram bacterial pellet and placed the tube with the solution for 20 min on a rolling shaker. To lyse the bacteria completely, each of the dissolved pellets was sonicated. We then centrifuged the solutions with 60,000 × g for 30 min. As our protein formed inclusion bodies, we discarded the supernatants and resuspended each pellet in lysis buffer (PBS, 1 mM DTT, 0.1 % Triton X-100, pH 7.4). Sonication, centrifugation and resuspension steps were repeated until the supernatants were clear solutions. Each pellet was then resuspended in a denaturation buffer (PBS, 6 M guanidine hydrochloride, pH 7.5), sonicated and centrifuged. We kept the supernatants and measured the absorption at 280 nm. The solutions were then mixed in a ratio of 1:10 (functional subunits with His-tag to non-functional subunits) according to the measured absorption. We slowly pipetted the mixture into 500 ml of refolding buffer (PBS, 10 mM β-mercaptoethanol, pH 7.4) and placed it on a magnetic stirrer for 15 h.

The solution was centrifuged at 14,000 × g for 10 min. The supernatant was filtered through a hydrophilic 0.22 μm MF-Millipore Membrane and loaded on a 5 ml HisTrap FF (GE Healthcare, Little Chalfont, UK) that had been equilibrated with binding buffer (PBS, 10 mM imidazole, pH 7.4). After washing the loaded column with binding buffer, the recovery of the protein was accomplished using a gradient elution (elution buffer: PBS, 250 mM imidazole, pH 7.4). The flow through was fractionated. Fractions were analyzed using absorption

spectroscopy and gel electrophoresis. Fractions containing SA were dialyzed against PBS and stored at 4 °C.

References

1. Baumann F, Bauer MS, Milles LF, Alexandrovich A, Gaub HE, Pippig DA. Monovalent Strep-Tactin for strong and site-specific tethering in nanospectroscopy. Nat Nanotechnol. 2016;11(1):89-94. doi: 10.1038/nnano.2015.231. PubMed PMID: 26457965.
