# Supplementary material for: Monodisperse measurement of the biotin-streptavidin interaction strength in a well-defined pulling geometry
Source: PLoS One. 2017 Dec 5;12(12):e0188722. doi: 10.1371/journal.pone.0188722 (PMC5716544; doi:10.1371/journal.pone.0188722)
Supplement: S2 Appendix — (PDF) [file pone.0188722.s002.pdf]

S2. Appendix.

Exemplary force-distance curves

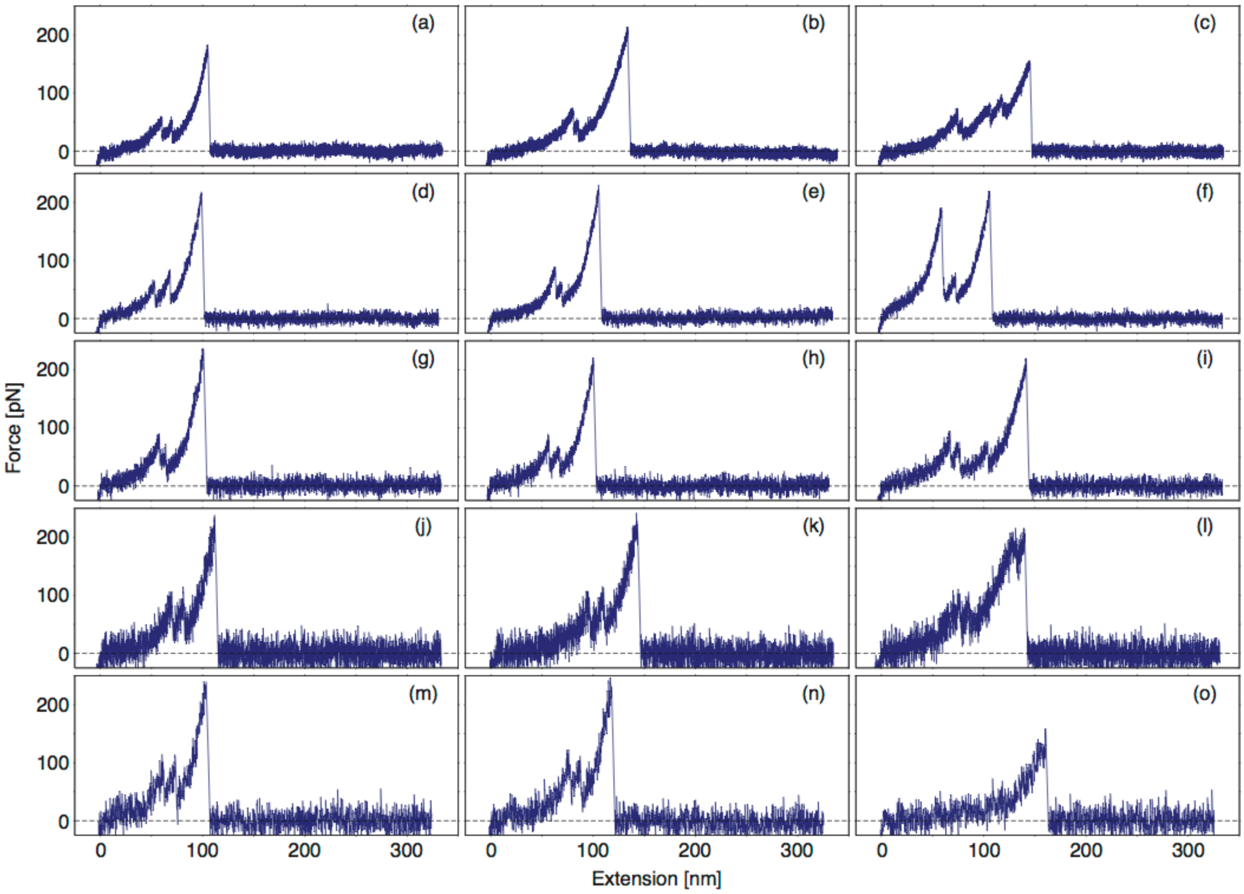

Fig A. Exemplary force-distance curves. Force-extension data recorded for different pulling velocities: (a-c) 200 nm/s, (d-f) 800 nm/s, (g-i) 2,000 nm/s, (j-l) 5,000 nm/s, and (m-o) 10,000 nm/s. The curves depicted in the left and in the middle column show a clear unfolding pattern of the calibration domain indicating specific single-molecule interaction. The curves depicted in the right column show interaction, but no clear unfolding pattern of the calibration domain is visible. These curves were thus not considered for further evaluation. The curves in (c), (i), and (l) are most probably caused by interaction of more than one biotin:mSA pair. For the curve in (f), a ddFLN4-like pattern is visible, but the unfolding force of the calibration domain is too high. Unspecific sticking of PEG or pulling with unfolded ddFLN4 may have caused the curve shown in (o).
