# Supplementary material for: Monodisperse measurement of the biotin-streptavidin interaction strength in a well-defined pulling geometry
Source: PLoS One. 2017 Dec 5;12(12):e0188722. doi: 10.1371/journal.pone.0188722 (PMC5716544; doi:10.1371/journal.pone.0188722)
Supplement: S3 Appendix — (PDF) [file pone.0188722.s003.pdf]

Long-term SMFS measurement

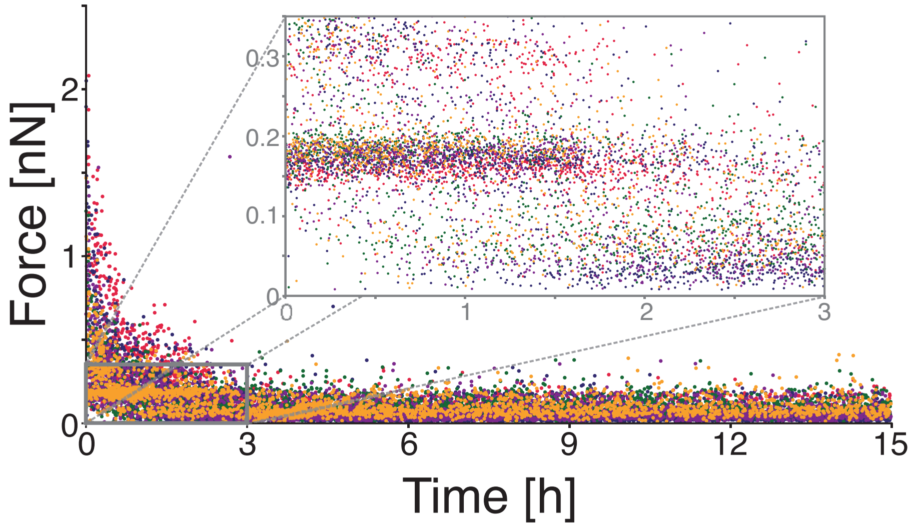

Fig A. Interaction of cantilever and surface over the course of the measurement. The force of the last peak in all force-extension curves that showed interaction between cantilever and surface are plotted over time. The different colors correspond to the different retraction velocities, with the color-coding being the same as in the main text. At the beginning of the measurement, multiple interactions give rise to high rupture forces. During the first 2.5 h (inset), a lot of specific single-molecule interactions are present resulting in a band of colored circles at about 200 pN. Wear out effects of cantilever and surface functionalization cause an increase of unspecific low-force interaction. For some of these, ddFLN4 unfolding is seen causing a small but broad unbinding peak at 100-160 pN in the histogram of rupture forces (Fig B).

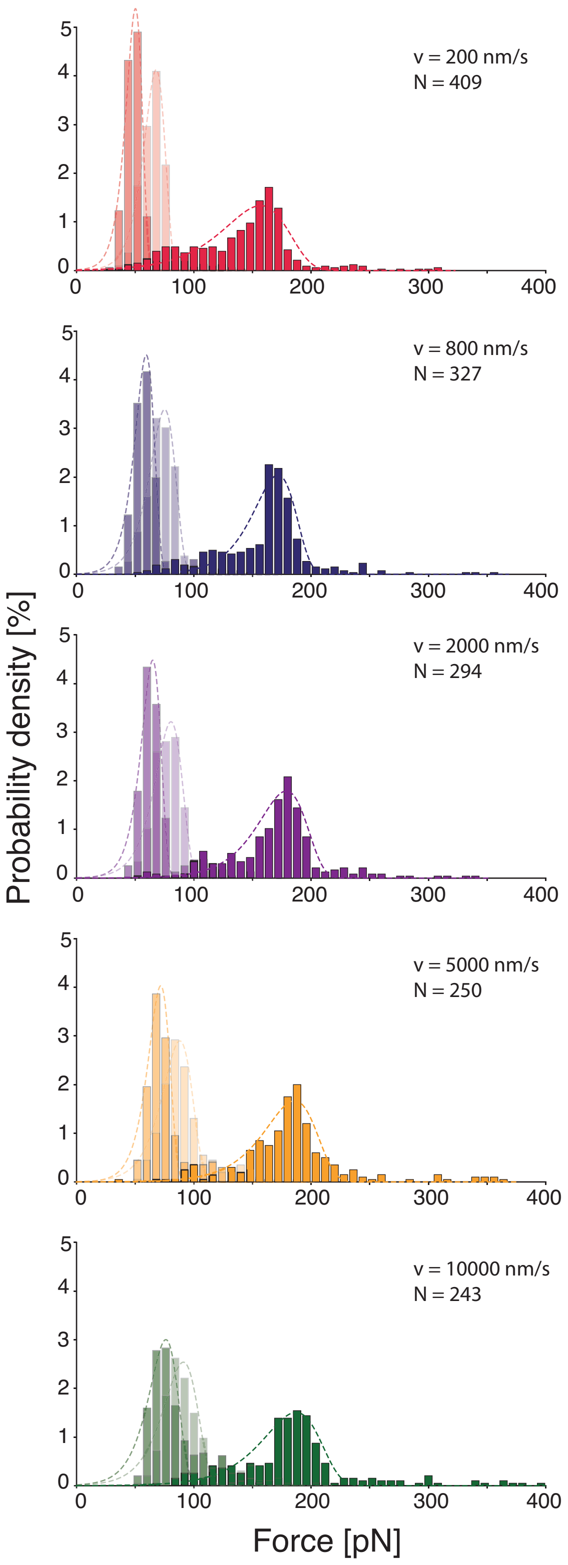

Fig B. Force histograms for a 15 h measurement. Unfolding and unbinding forces are plotted in the same manner as for Figure 6. For this experiment, the spring constant of the cantilever was 53 pN/nm. For this measurement, a second peak at lower forces is visible for the unbinding forces. From the course of the measurement (Fig A), it is obvious that the amount of low unbinding forces is insignificant in the first 2.5 h of the measurement. Therefore, the second peak cannot be caused by different binding states of biotin and mSA. The absence of a second binding state is further substantiated by the fact that for the lower unbinding forces, the unfolding forces of ddFLN4 are not shifted towards lower forces. As suggested by Schoeler et al. [1], such a bias occurs if there is an overlap of the probability distributions corresponding to unfolding and unbinding. Since they mostly occur for the slow retraction velocities, i.e. for long surface contact, we attribute these low unbinding forces to unspecific sticking of the cantilever to the surface resulting in ddFLN4 like force-extension patterns. Specific interaction at high forces was yet still detectable after 15 h of continuous measurement at room temperature. The time scale for the undisturbed interaction, i.e. without the additional low unbinding forces, is still sufficient for all immobilization and labeling applications of mSA envisioned in the main text.

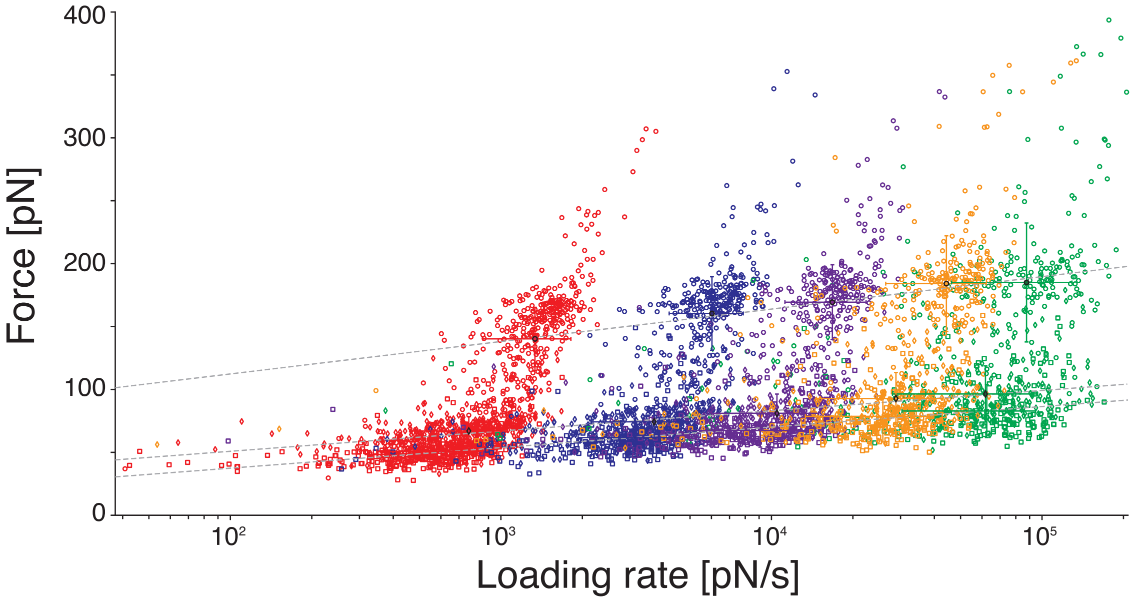

Fig C. Bell-Evans plot for a 15 h measurement. Data and color-coding are the same as in Fig B. Unfolding forces of ddFLN4 are plotted with open squares and diamonds, unbinding forces for biotin:mSA with open circles. Dashed lines are linear fits to the centers of gravity (shown as filled circles and diamonds) of the distributions of forces and loading rates, respectively. Colored crosses indicate the corresponding standard deviations. We find $\Delta x_0 = (0.59 \pm 0.06)$ nm and $k_{off,0} = 1 \times 10^{-2}$ s^{-1} for the first unfolding step of ddFLN4, $\Delta x_0 = (0.58 \pm 0.04)$ nm and $k_{off,0} = 7 \times 10^{-2}$ s^{-1} for the second unfolding step of ddFLN4, and $\Delta x_0 = (0.37 \pm 0.03)$ nm and $k_{off,0} = 4 \times 10^{-4}$ s^{-1} for the rupture of the biotin:mSA-complex.

References

1. Schoeler C, Verdorfer T, Gaub HE, Nash MA. Biasing effects of receptor-ligand complexes on protein-unfolding statistics. Phys Rev E. 2016;94(4-1):042412. doi: 10.1103/PhysRevE.94.042412. PubMed PMID: 27841541.
