# Supplementary material for: Monodisperse measurement of the biotin-streptavidin interaction strength in a well-defined pulling geometry
Source: PLoS One. 2017 Dec 5;12(12):e0188722. doi: 10.1371/journal.pone.0188722 (PMC5716544; doi:10.1371/journal.pone.0188722)
Supplement: S4 Appendix — (PDF) [file pone.0188722.s004.pdf]

S4 Appendix.

Estimating the contour lengths of PEG and ddFLN4

In our experiments we use polyethylene glycol with a molecular weight of 5,000 g/mol. The molar mass of PEG is given by (18.02 + 44.05 × n) g/mol, where n is the number of subunits. For PEG5000, the number of subunits is $n = 113$. The net length of a segment is reported to be in the range of 0.278 nm to 0.358 nm depending on the orientation of the bonds [1]. We thus estimate the contour length of a PEG5000 polymer to be in the range of 31 nm to 40 nm. In this estimation, *N*-Hydroxysuccinimide and maleimide are not considered.

Our ddFLN4 consists of 101 amino acids. Assuming a length of 0.36 nm per amino acid, the contour length of the pure ddFLN4 reads 36 nm. We are neither taking into account additional length caused by linkers nor are we correcting for the end-to-end-distance of the folded ddFLN4, when considering the contour length increment upon unfolding.

References

1. Oesterhelt F, Rief M, Gaub HE. Single molecule force spectroscopy by AFM indicates helical structure of poly(ethylene-glycol) in water. New Journal of Physics. 1999;1(1):6.
