# Supplementary material for: Monodisperse measurement of the biotin-streptavidin interaction strength in a well-defined pulling geometry
Source: PLoS One. 2017 Dec 5;12(12):e0188722. doi: 10.1371/journal.pone.0188722 (PMC5716544; doi:10.1371/journal.pone.0188722)
Supplement: S5 Appendix — (PDF) [file pone.0188722.s005.pdf]

S5 Appendix.

Formulas

Bell-Evans distribution

$$p(F) = \frac{k_{off,0}}{\dot{F}} \cdot e^{\frac{F\Delta x_0}{k_B T}} \cdot e^{(\frac{k_{off,0}}{\dot{F}}\frac{k_B T}{\Delta x_0}(1-e^{\left(\frac{F\Delta x_0}{k_B T}\right)}))}$$

Loading-rate ($\dot{F}$) dependence of unbinding or unfolding force (F^*)

$$F^*(\dot{F}) = \frac{k_B T}{\Delta x_0}\log(\frac{\dot{F}}{k_{off,0}}\frac{\Delta x_0}{k_B T})$$

Worm-like chain model

$$F(x) = \frac{kT}{4p}\left(\left(1-\frac{x}{l}\right)^{-2} - 1 + 4\frac{x}{l}\right)$$

Transformation into contour length space

$$L(F,x) = Re\left(\frac{xkT}{6Fp}\left(3 + 4\frac{Fp}{kT} + \frac{4\left(\frac{Fp}{kT}\right)^2 - 3\frac{F}{kT} + 9}{f(kT,F,p)} + f(kT,F,p)\right)\right)$$

$$f(kT,F,p) = \left(27 - \frac{27Fp}{2kT} + \left(6\frac{Fp}{kT}\right)^2 - \left(2\frac{Fp}{kT}\right)^3 + \frac{3}{2}\sqrt{-3\left(\frac{Fp}{kT}\right)^2\left(\left(4\frac{Fp}{kT} - 3\right)^3 - 108\right)}\right)^{\frac{1}{3}}$$
