# Supplementary material for: Monodisperse measurement of the biotin-streptavidin interaction strength in a well-defined pulling geometry
Source: PLoS One. 2017 Dec 5;12(12):e0188722. doi: 10.1371/journal.pone.0188722 (PMC5716544; doi:10.1371/journal.pone.0188722)
Supplement: S6 Appendix — (PDF) [file pone.0188722.s006.pdf]

S6 Appendix.

Fitted Bell-Evans distributions shown in Fig 6

**First ddFLN4 unfolding peak**					
v **[nm/s]**	**200**	**800**	**2,000**	**5,000**	**10,000**
$\dot{F}$ **[pN/s]**	768	3,519	10,080	29,010	66,710
F **[pN]**	66	75	83	89	90
Δx_0 **[nm]**	0.56	0.47	0.37	0.42	0.42
$k_{off,0}$ **[s^{-1}]**	7×10^{-3}	7×10^{-2}	6×10^{-1}	4×10^{-1}	7×10^{-1}
**Second ddFLN4 unfolding peak**					
v **[nm/s]**	**200**	**800**	**2,000**	**5,000**	**10,000**
$\dot{F}$ **[pN/s]**	701	3,609	9,841	29,820	76,030
F **[pN]**	59	70	78	87	94
Δx_0 **[nm]**	0.47	0.50	0.41	0.42	0.34
$k_{off,0}$ **[s^{-1}]**	1×10^{-1}	9×10^{-2}	4×10^{-1}	5×10^{-1}	2
**Biotin:mSA unbinding peak**					
v **[nm/s]**	**200**	**800**	**2,000**	**5,000**	**10,000**
$\dot{F}$ **[pN/s]**	1,736	7,469	20,680	52,390	111,900
F **[pN]**	201	212	217	222	230
Δx_0 **[nm]**	0.33	0.35	0.37	0.28	0.22
$k_{off,0}$ **[s^{-1}]**	2×10^{-5}	9×10^{-6}	8×10^{-6}	1×10^{-3}	2×10^{-1}

Table A. Fitted Bell-Evans distributions shown in Fig 6. To the histograms shown in Fig 6, Bell-Evans distributions were fitted. Mean loading rate used for the fit, most probable rupture force determined from the fit, and fitting parameters (distance to transition state and zero-force off-rate) are listed for the five retraction velocities and the different force peaks.
