# Supplementary material for: Monodisperse measurement of the biotin-streptavidin interaction strength in a well-defined pulling geometry
Source: PLoS One. 2017 Dec 5;12(12):e0188722. doi: 10.1371/journal.pone.0188722 (PMC5716544; doi:10.1371/journal.pone.0188722)
Supplement: S7 Appendix — (PDF) [file pone.0188722.s007.pdf]

S7 Appendix.

Sequences of protein constructs

Functional core SA subunit with an N-terminal His-tag (green) and a unique cysteine (cyan):

MGSSHHHHHHMCGSEAGITGTWYNQLGSTFIVTAGADGALTGTYESAVGNAESRY
VLTGRYDSAPATDGSGTALGWTVAWKNNYRNAHSATTWSGQYVGGAEARINTQW
LLTSGTTEANAWKSTLVGHDTFTKVKPSAAS

Functional core SA subunit:

MEAGITGTWYNQLGSTFIVTAGADGALTGTYESAVGNAESRYVLTGRYDSAPATDG
SGTALGWTVAWKNNYRNAHSATTWSGQYVGGAEARINTQWLLTSGTTEANAWKS
TLVGHDTFTKVKPSAAS

Non-functional core SA subunit with three mutations (red; N23A, S27D, S45A):

MEAGITGTWYAQLGDTFIVTAGADGALTGTYEAAVGNAESRYVLTGRYDSAPATDG
SGTALGWTVAWKNNYRNAHSATTWSGQYVGGAEARINTQWLLTSGTTEANAWKS
TLVGHDTFTKVKPSAAS

YbbR-tagged (magenta) ddFLN4 construct with N-terminal His-tag (green) and C-terminal cysteine (cyan). A cysteine that could potentially be accessible for binding to maleimide was mutated to serine (red; C18S):

MDSLEFIASKLAHHHHHHGSADPEKSYAEGPGLDGGESFQPSKFKIHAVDPDGVHRT
DGGDGFVVTIEGPAPVDPVMVDNGDGTYDVEFEPKEAGDYVINLTLDGDNVNGFPK
TVTVKPAPGSC

YbbR-tagged (yellow) superfolder GFP construct with N-terminal His-tag (green) and cysteine (cyan) for tethering. A cysteine that could potentially be accessible for binding to maleimide was mutated to serine (red; C48S):

MGSSHHHHHHLEVLFQGPGHMCGSGSMSKGEELFTGVVPILVELDGDVNGHKFSVR
GEGEGDATIGKLTLKFISTTGKLPVPWPTLVTTLTYGVQCFSRYPDHMKRHDFFKSA
MPEGYVQERTISFKDDGKYKTRAVVKFEGDTLVNRIELKGTDFKEDGNILGHKLEYN
FNSHNVYITADKQKNGIKANFTVRHNVEDGSVQLADHYQQNTPIGDGPVLLPDNHY
LSTQTVLSKDPNEKRDHMVLHEYVNAAGITHGMDELYKSGSGSASDSLEFIASKLA
