# Supplementary material for: Monodisperse measurement of the biotin-streptavidin interaction strength in a well-defined pulling geometry
Source: PLoS One. 2017 Dec 5;12(12):e0188722. doi: 10.1371/journal.pone.0188722 (PMC5716544; doi:10.1371/journal.pone.0188722)
Supplement: S8 Appendix — (PDF) [file pone.0188722.s008.pdf]

Measuring with mSA immobilized on the cantilever

To test the stability of mSA as an anchor for SMFS, we also performed measurements in the opposite configuration, i.e. attaching mSA to the cantilever and biotinylated proteins to the surface (Figures S5 and S6). In this configuration, refolding of the control domain is unnecessary, because for every force-distance curve a new calibration domain is available on the surface. We used biotinylated GFP, whose unfolding pattern is well characterized [1], as calibration domain.

For these measurements, the distribution of rupture forces is much broader and slightly shifted to lower forces compared to the measurements with mSA on the surface. As we find the same effect, when immobilizing biotinylated ddFLN4 on the surface, we suspect shift and broadening of the distributions to be caused by slow degradation of the mSA molecules on the cantilever. This could imply that in this specific pulling geometry unbinding of biotin involves partial unfolding of the functional mSA subunit. To probe this hypothesis, steered molecular dynamics simulations could be helpful, but this is beyond the scope of this study.

S8 Appendix.

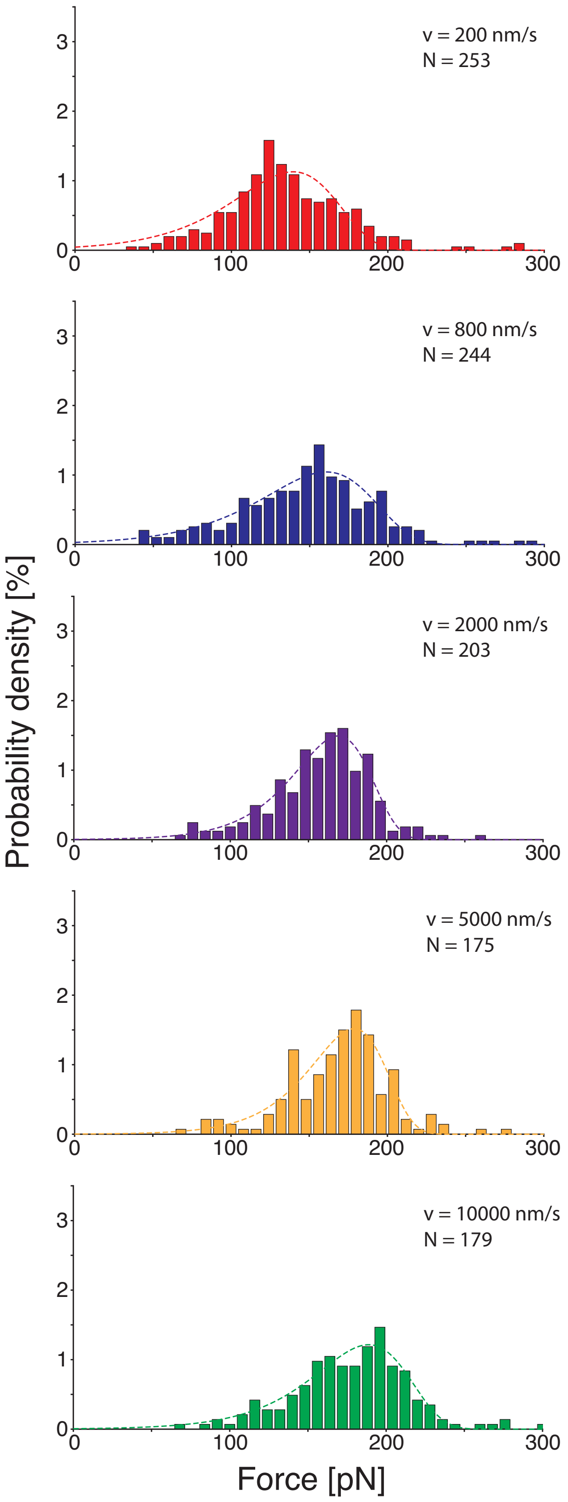

Fig A. Force Histograms, when measuring with mSA immobilized on the cantilever. For this measurement, mSA was immobilized on the cantilever and biotinylated GFP was attached to the surface. The spring constant of the cantilever was $k = 69.8$ pN/nm. The dashed lines show independent fits of Bell-Evans distributions to the force histograms.

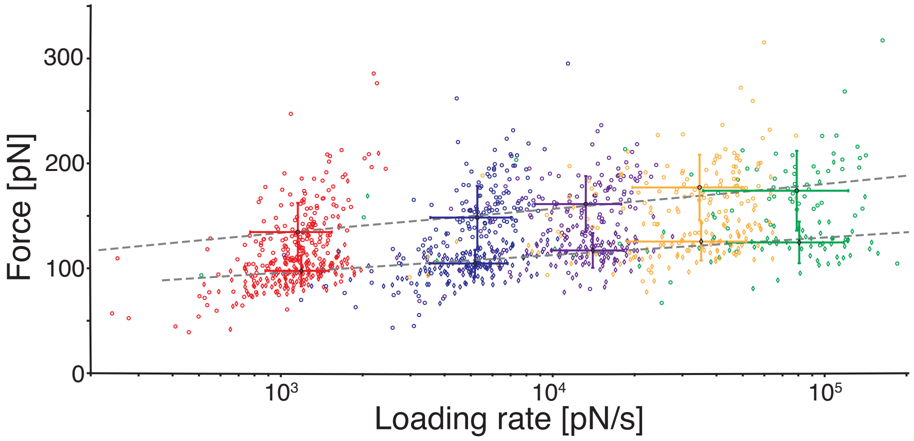

Fig B. Dynamic Force Spectrum for unfolding of GFP and unbinding of biotin:mSA. Color-coding is the same as in Fig A. Unfolding forces of GFP are plotted with open diamonds, rupture forces of the complex with open circles. Dashed lines are linear fits to the centers of gravity (shown as filled circles and diamonds) of the distributions of forces and loading rates, respectively. Colored crosses indicate the corresponding standard deviations. We find $\Delta x_0 = (0.56 \pm 0.08)$ nm and $k_{off,0} = 2 \times 10^{-4}$ s^{-1} for the unfolding of GFP and $\Delta x_0 = (0.39 \pm 0.05)$ nm and $k_{off,0} = 3 \times 10^{-4}$ s^{-1} for the rupture of the biotin:mSA-complex.

References

1. Dietz H, Rief M. Exploring the energy landscape of GFP by single-molecule mechanical experiments. Proc Natl Acad Sci U S A. 2004;101(46):16192-7. doi: 10.1073/pnas.0404549101. PubMed PMID: 15531635; PubMed Central PMCID: PMCPMC528946.
